# Direct observation of epoxy resin blocks for renal biopsy by low-vacuum scanning electron microscopy

**DOI:** 10.1007/s00795-023-00356-x

**Published:** 2023-05-10

**Authors:** Akihiro Tojo, Makoto Abe, Kin-ichi Matsuyama

**Affiliations:** 1 Department of Nephrology and Hypertension, Dokkyo Medical University, 880 Kitakobayashi, Mibu, Tochigi 321-0293 Japan; 2 Department of Diagnostic Pathology, Dokkyo Medical University, Mibu, Tochigi Japan

**Keywords:** Renal biopsy, Electron microscopy, LVSEM, Glomerulus, Podocyte

## Abstract

To improve the resolution of low-vacuum scanning electron microscopy (LVSEM), the epoxy resin block for the transmission electron microscopy (TEM) was observed directly with LVSEM. After observing ultrathin sections from renal biopsies of IgA nephropathy, membranous nephropathy, lupus nephritis, diabetic nephropathy (DM), thin basement membrane disease (TBMD), Alport’s syndrome, Fabry’s disease, and renal amyloidosis, the epoxy resin blocks of the same sites were observed by LVSEM and compared. The LVSEM image of the epoxy resin block corresponds to the negative of the TEM image, and when the gradation is reversed, the LVSEM image was comparable to the TEM image. At a low magnification of 100 ×, the entire specimen, including the glomerulus, was obtained. LVSEM at 5000 × magnification was sufficient to identify paramesangial deposits in IgA nephropathy and subepithelial electron-dense deposits (EDD) and spikes in membranous nephropathy. Glomerular basement membrane thickening in DM and thinning in TBMD could be sufficiently diagnosed with LVSEM at 6000 ×. Accumulation of ceramide in Fabry's disease was easily identified, but amyloid fibril could not be identified by LVSEM. LVSEM of renal biopsy epoxy resin blocks can replace TEM up to moderate magnification.

## Introduction

An electron microscope is essential for renal biopsy, but more than 3 days and advanced technology are required to obtain images. Therefore, low-vacuum scanning electron microscopy (LVSEM), which can observe periodic acid methenamine silver (PAM)-stained optical microscope glass samples in a wet state, was expected to be an alternative to TEM because specimens can be easily and quickly observed [1, 2]. Platinum blue staining was performed to improve resolution [1, 3, 4], but ultrafine LVSEM images were inferior to transmission electron microscopy (TEM) images because light microscope (LM) samples for LVSEM observation had undergone paraffin embedding, deparaffinization, and staining processes. In this study, to overcome the limitations of optical microscope specimens, epoxy resin blocks were directly observed by LVSEM, and the results were compared with those from TEM. We investigated the efficacy of LVSEM for renal biopsy diagnosis in representative renal diseases, such as IgA nephropathy, membranous nephropathy, lupus nephritis, minimal change TBMD, Alport syndrome, Fabry disease, and renal amyloidosis.

## Materials and methods

**Fig. 1:**
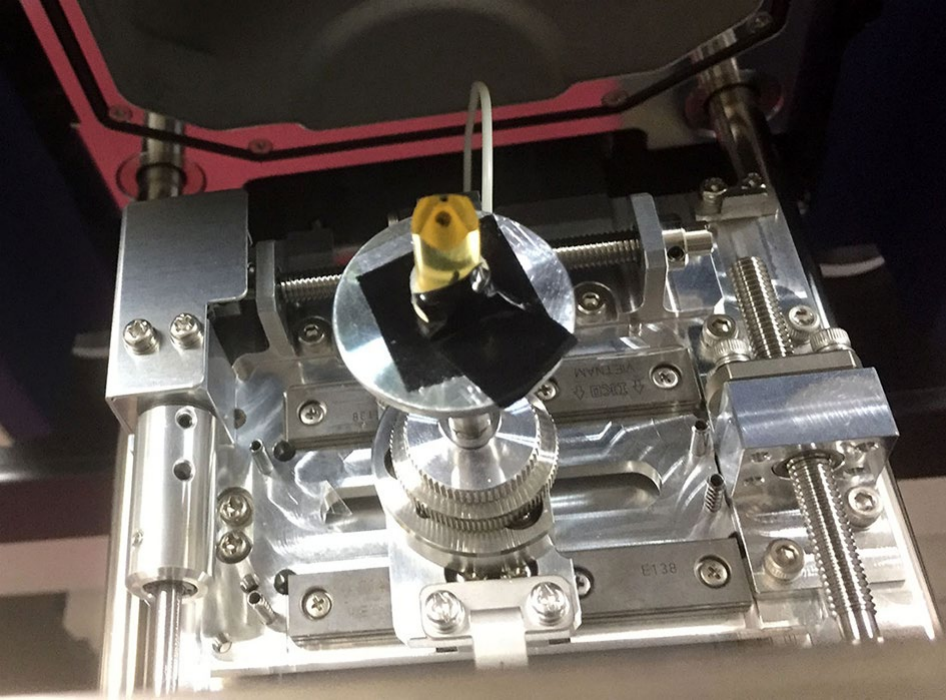
The epoxy resin block was placed on the sample stage of a low-vacuum scanning electron microscope (LVSEM) attached to a carbon tape

The epoxy resin block was placed on a sample stage with carbon tape (Fig. 1). It was observed with an LVSEM, TM4000Plus (Hitach High-Tech Co., Tokyo, Japan), at an accelerating voltage of 10–15 kV, as we have previously reported [2, 5, 6]. For usual TEM observation, ultrathin sections were cut from epoxy resin blocks of renal biopsy specimens, placed on 50-mesh copper grids, and examined with an HT7800 (Hitachi High-Tech). The LVSEM image has the same gradation as the negative in the TEM, and the gradation reversal to the image was performed with Adobe Photoshop 2023 (Adobe Systems Incorporated CA, USA) to obtain an image homologous to the TEM print images. In the present study, we observed samples from representative diseases, such as IgA nephropathy, membranous nephropathy, lupus nephritis, minimal change group, thin basement membrane disease (TBMD), Alport syndrome, Fabry disease, and renal amyloidosis. This study was conducted with consent for the use of renal biopsy specimens and ethics committee approval (R-3b-3J).

## Results

**Fig. 2:**
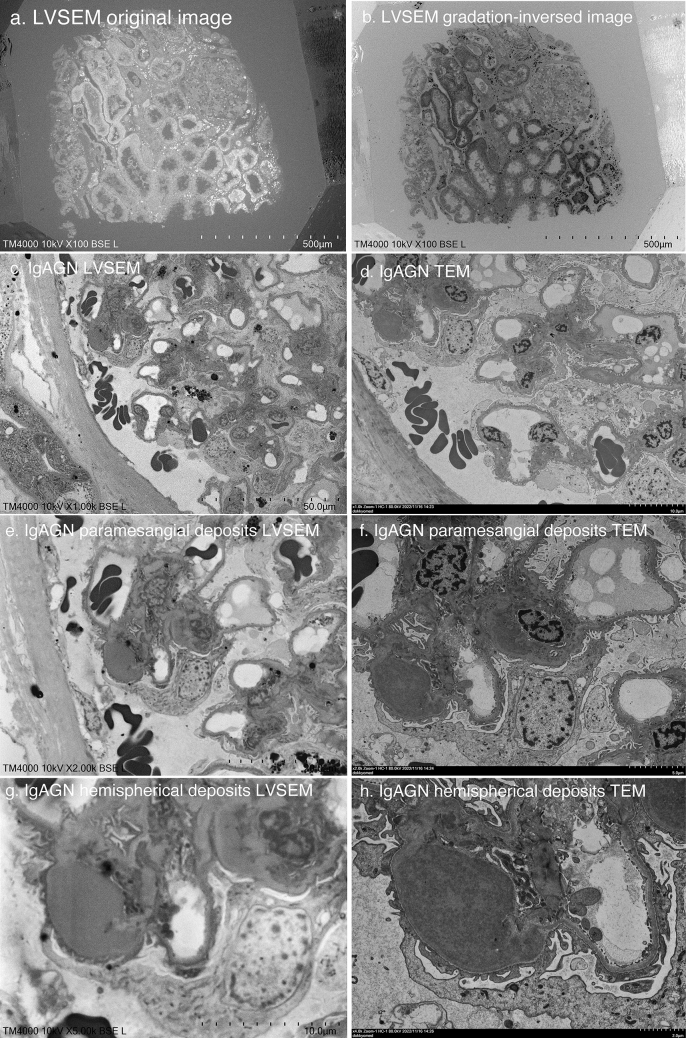
LVSEM images of an epoxy resin block of a renal biopsy sample of IgA nephropathy (**a**, **b**, **c**, **e**, **g**), and transmission electron microscopy (TEM) images of ultrathin sections from the same epoxy resin block (**d**, **f**, **h**)

LVSEM images of epoxy resin blocks were compared with TEM images of IgA nephropathy, which is most frequently observed in renal biopsies (Fig. 2). When the epoxy resin block was observed with LVSEM, the renal tubules appeared white and negative, but after gradation inversion, they became similar to the TEM image (Fig. 2a, b). First, ultrathin sections were taken by TEM at stepped magnifications of 1000 ×, 2000 × and 4000 × (Fig. 2d, f, h), and then the epoxy resin block was observed by LVSEM. Comparing the TEM image and the LVSEM image, we were able to observe the same site. However, there was some deviation due to sectioning. Even when taken at the same magnification, the LVSEM images (Fig. 2c, e, g) had a wider field of view and were smaller than the TEM images (Fig. 2d, f, h). Paramesangial electron-dense deposits can be observed in LVSEM images, which is consistent with the TEM diagnosis of IgA nephropathy. The density of the electron-dense deposits is slightly lower and flatter in the LVSEM image compared to the TEM image.

**Fig. 3:**
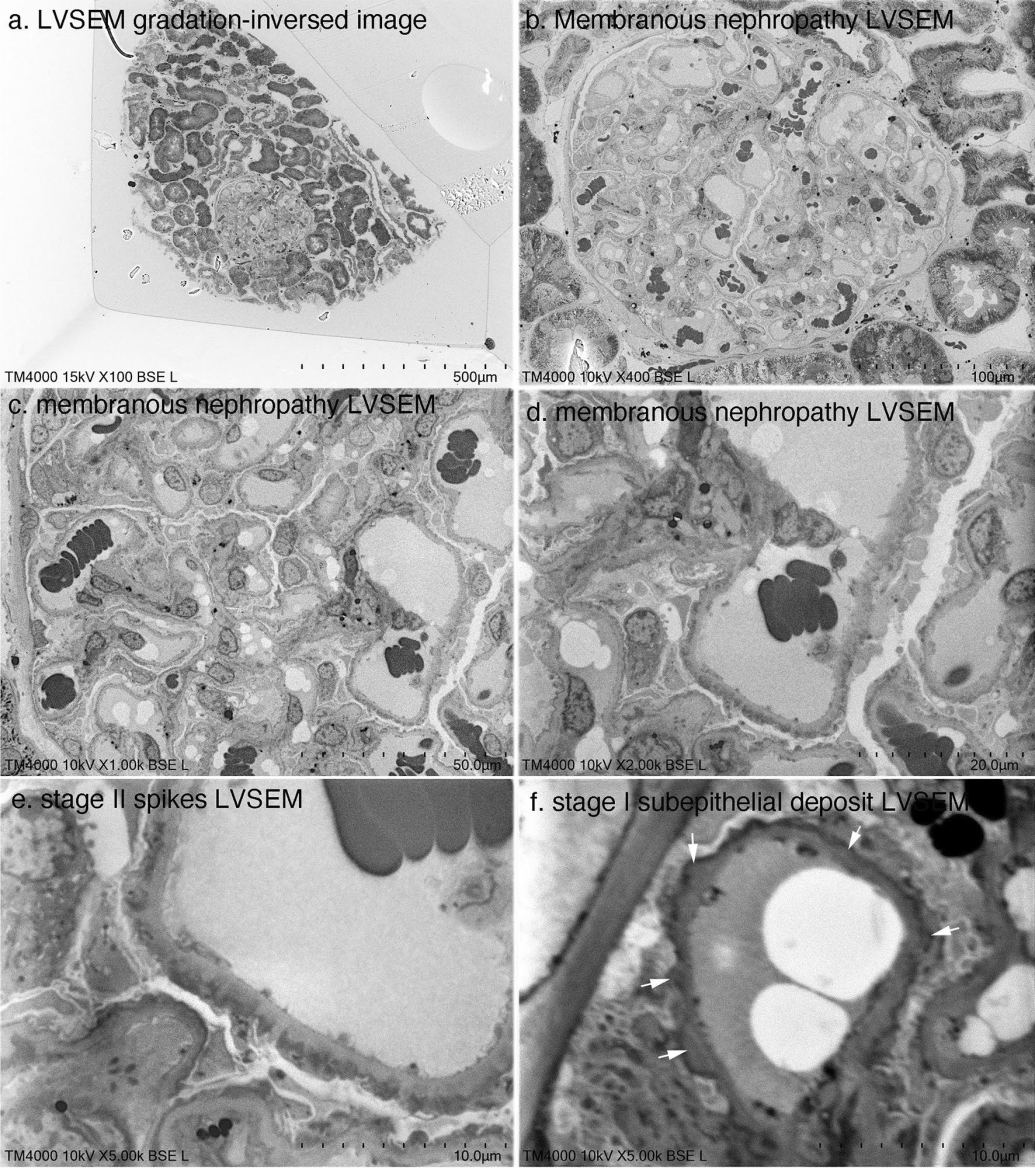
LVSEM images of an epoxy resin blocks of a renal biopsy sample of stage II (**a**–**e**) and stage I (**f**) membranous nephropathy. The arrows indicate subepithelial electron-dense deposits

**Fig. 4:**
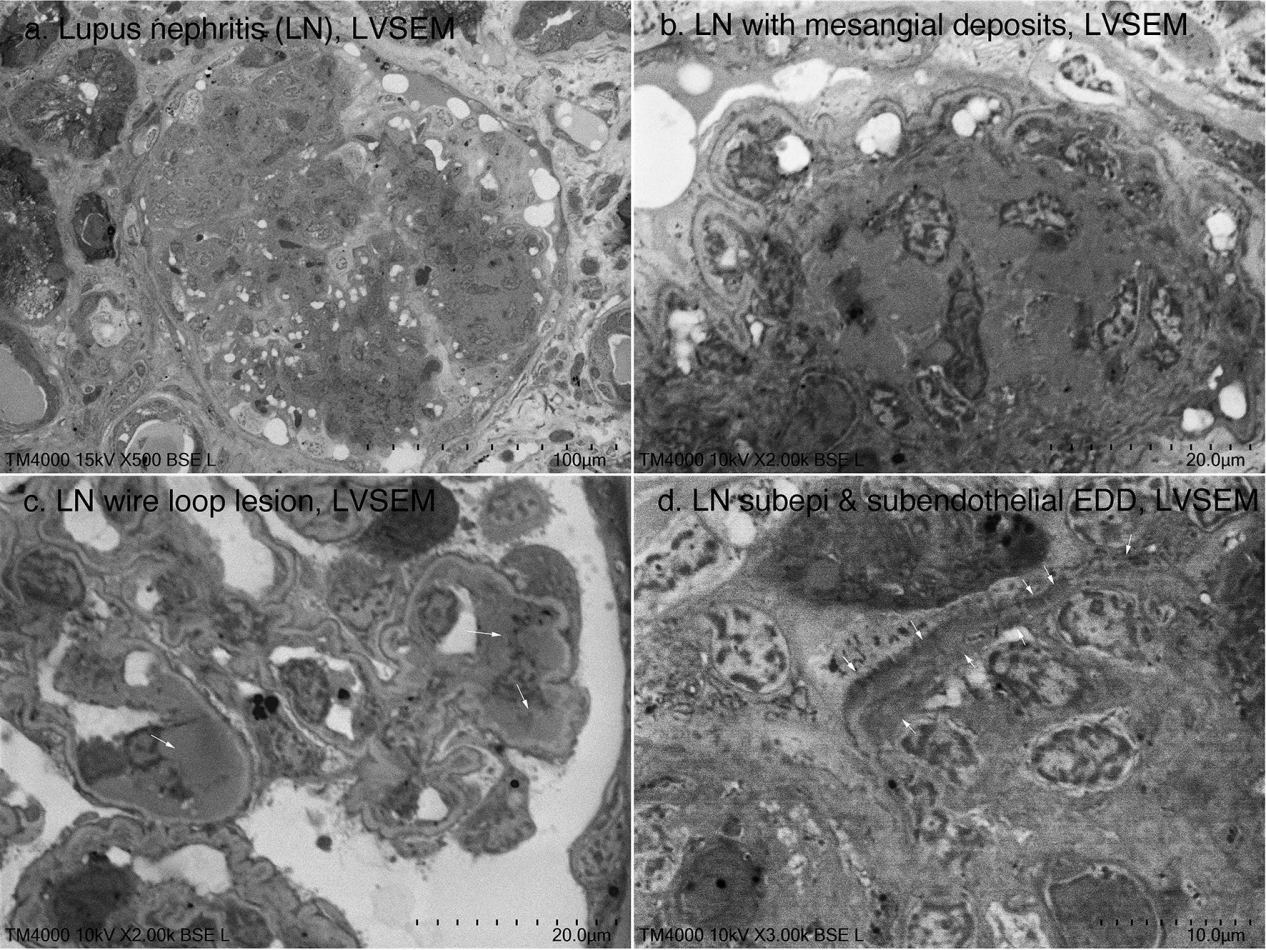
LVSEM images of an epoxy resin block of a renal biopsy sample of lupus nephritis. The arrows indicate wire loop lesion in **c**, and subendothelial and subepithelial electron-dense deposits in **d**

Then, the magnification was gradually increased from 100 × to 5000 × to determine whether membranous nephropathy could be diagnosed by LVSEM alone (Fig. 3). Although the resolution of direct epoxy resin block observation by LVSEM was poor at more than 5000 × magnification, spikes in stage II (Fig. 3e) and subepithelial electron-dense deposits in stage I (Fig. 3f) were identified, which were sufficient for the diagnosis of membranous nephropathy on its own (Fig. 3). Next, we attempted to diagnose lupus nephritis by LVSEM alone. Lobular structures and mesangial proliferation with mesangial electron-dense deposits were observed by LVSEM, but it was difficult to distinguish them from matrix increases (Fig. 4a, b). Wire loop lesions (Fig. 4c), double contour of the basement membrane, and subendothelial/subepithelial electron-dense deposits (EDD) (Fig. 4d) were observed, and lupus nephritis can be diagnosed by combining these findings with light microscope findings and immunofluorescence findings.

**Fig. 5:**
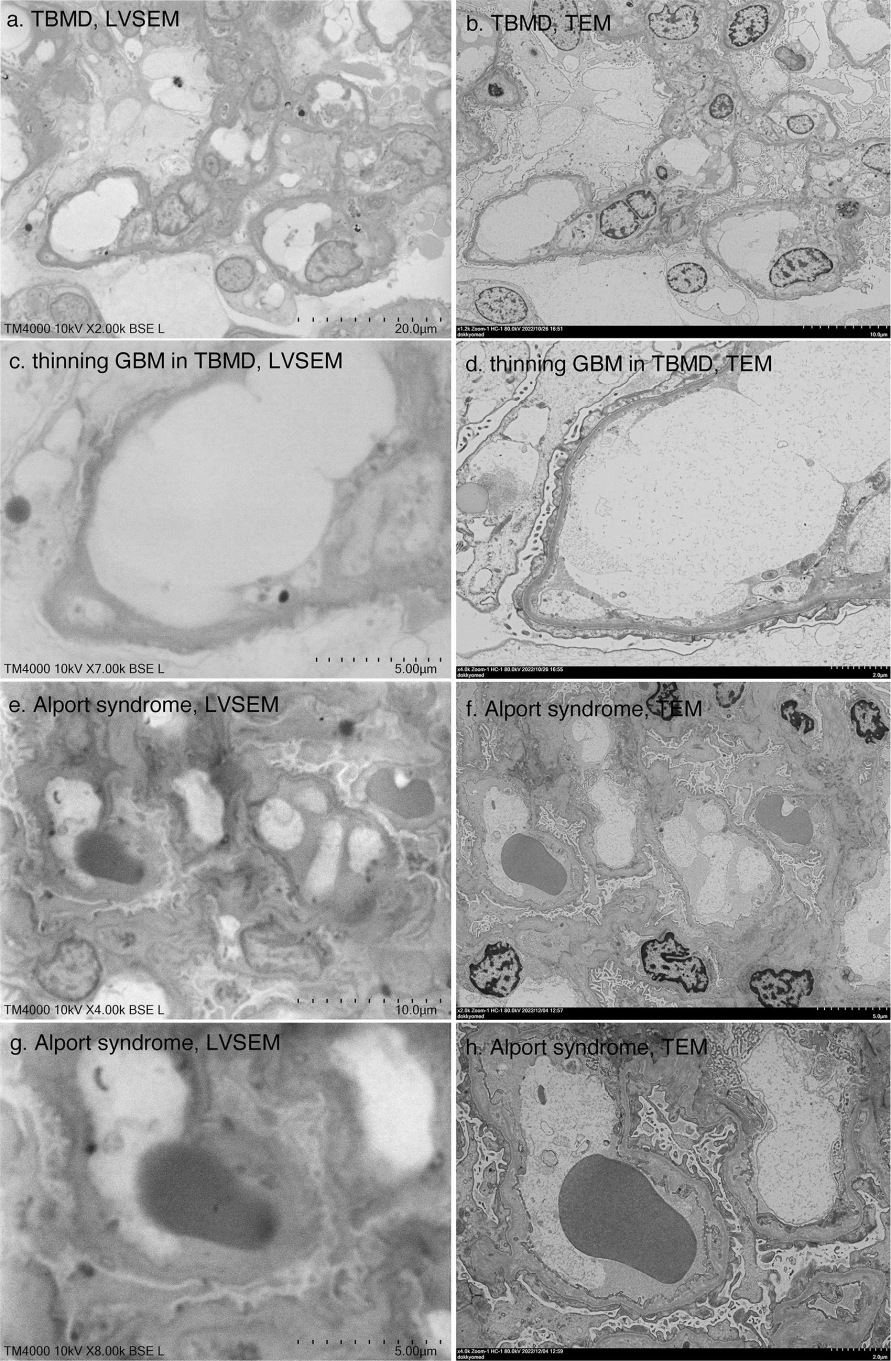
Comparison of LVSEM images of epoxy resin blocks (**a**, **c**, **e**, **g**) and TEM images of ultrathin sections (**b**, **d**, **f**, **h**) of the same area of renal biopsy samples of thin basement membrane disease (**a**–**d**) and in Alport syndrome (**e**–**h**)

Then, we compared LVSEM and TEM images of thin basement membrane syndrome and Alport syndrome samples to clarify the ability of LVSEM to distinguish changes in the glomerular basement membrane (GBM) (Fig. 5). LVSEM has poor resolution for distinguishing the three-layered structure of the GBM, namely, lamina rara interna, lamina densa, and lamina rara externa, at 7000 × magnification. Thus, the thickness of the basement membrane was estimated to be 225 nm by LVSEM (Fig. 5c), which was slightly thicker than the 180 nm estimated by TEM (Fig. 5d). However, a diagnosis of TBMD was possible. GBM thinning and thickening in Alport syndrome can be determined by LVSEM (Fig. 5e, g), whereas lamellation and reticular formation were only determined by TEM (Fig. 5f, h). Although the resolution of LVSEM images is not sufficient for diagnosis of Alport syndrome, changes in GBM by LVSEM images support clinicopathologic diagnosis.

**Fig. 6:**
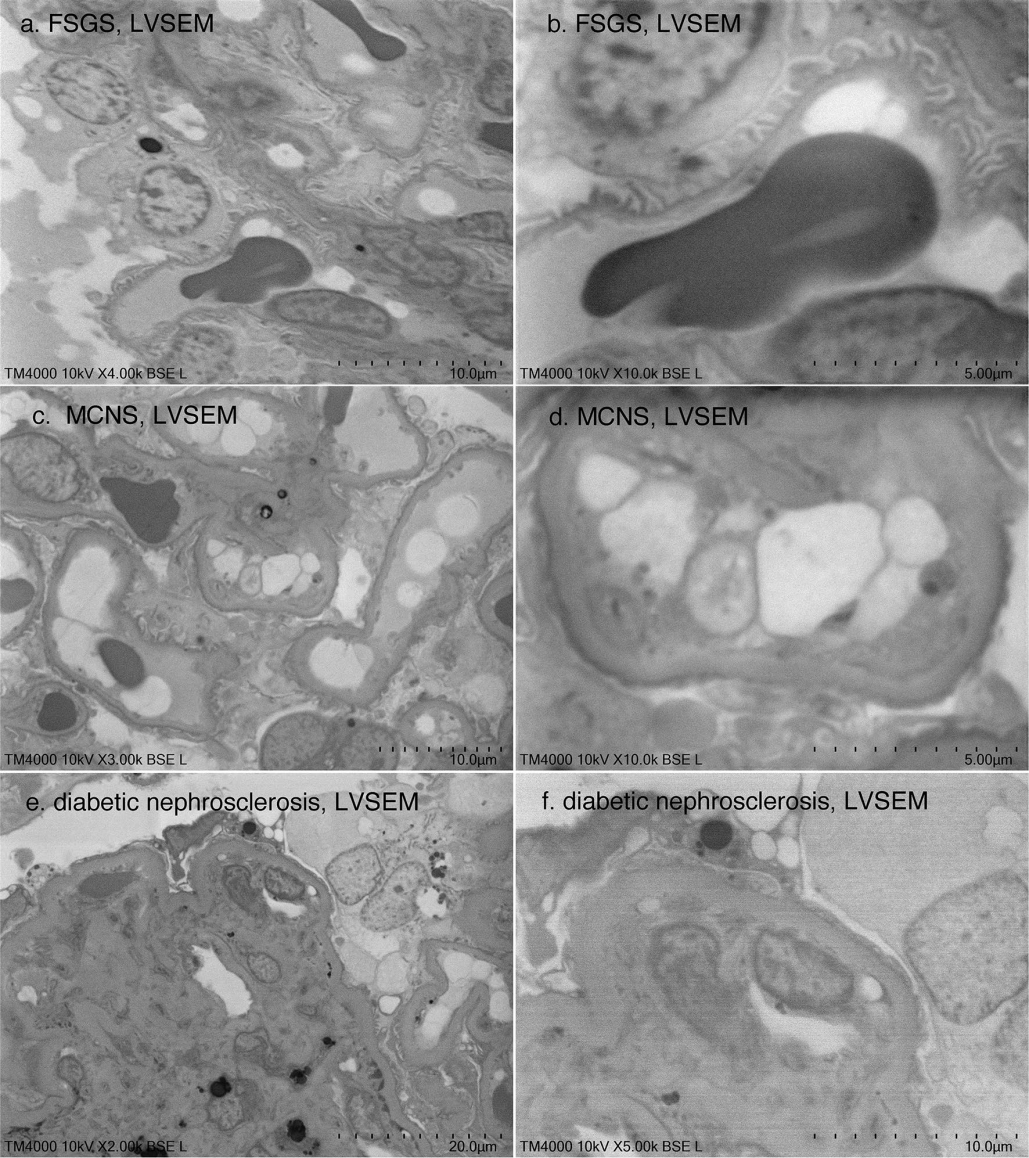
LVSEM images of epoxy resin blocks of renal biopsy samples with focal segmental glomerulosclerosis (FSGS) (**a**, **b**), minimal change nephrotic syndrome (MCNS) (**c**, **d**), and diabetic nephrosclerosis (**e**, **f**)

Ultrastructural changes in podocytes were assessed with direct observation of epoxy resin blocks by LVSEM. In FSGS, foot processes were relatively preserved even when nephrotic syndrome was observed (Fig. 6a, b), whereas in MCNS, foot process effacement was diffusely observed (Fig. 6c, d). In nephrotic diabetic nephrosclerosis, increased mesangial matrix, basement membrane thickening, and foot process effacement were clearly observed by LVSEM (Fig. 6e, f).

**Fig. 7:**
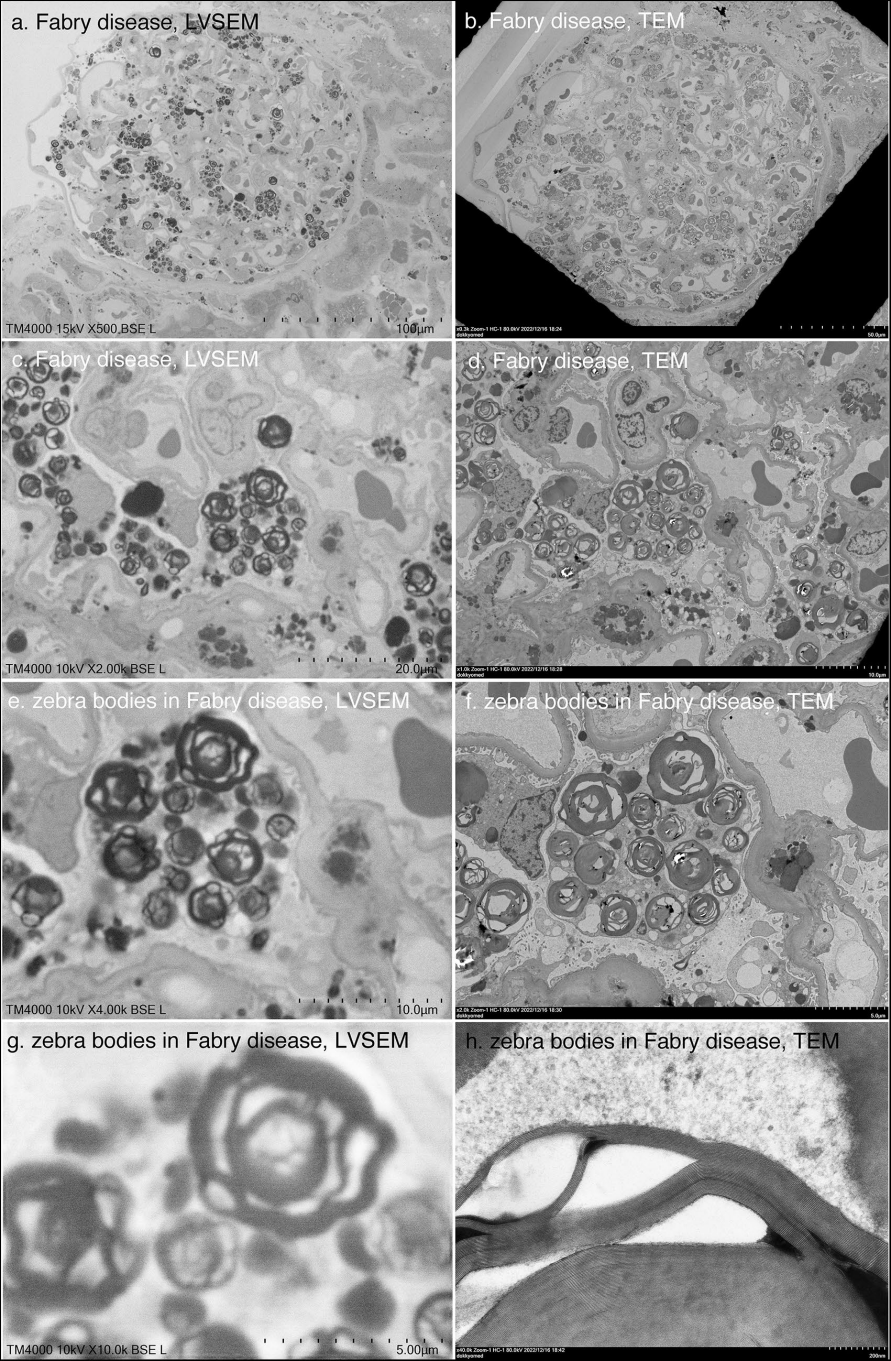
Comparison of LVSEM images of epoxy resin block (**a**, **c**, **e**, **g**) and TEM images of ultrathin section (**b**, **d**, **f**, **h**) of the same area of renal biopsy samples of Fabry disease

**Fig. 8:**
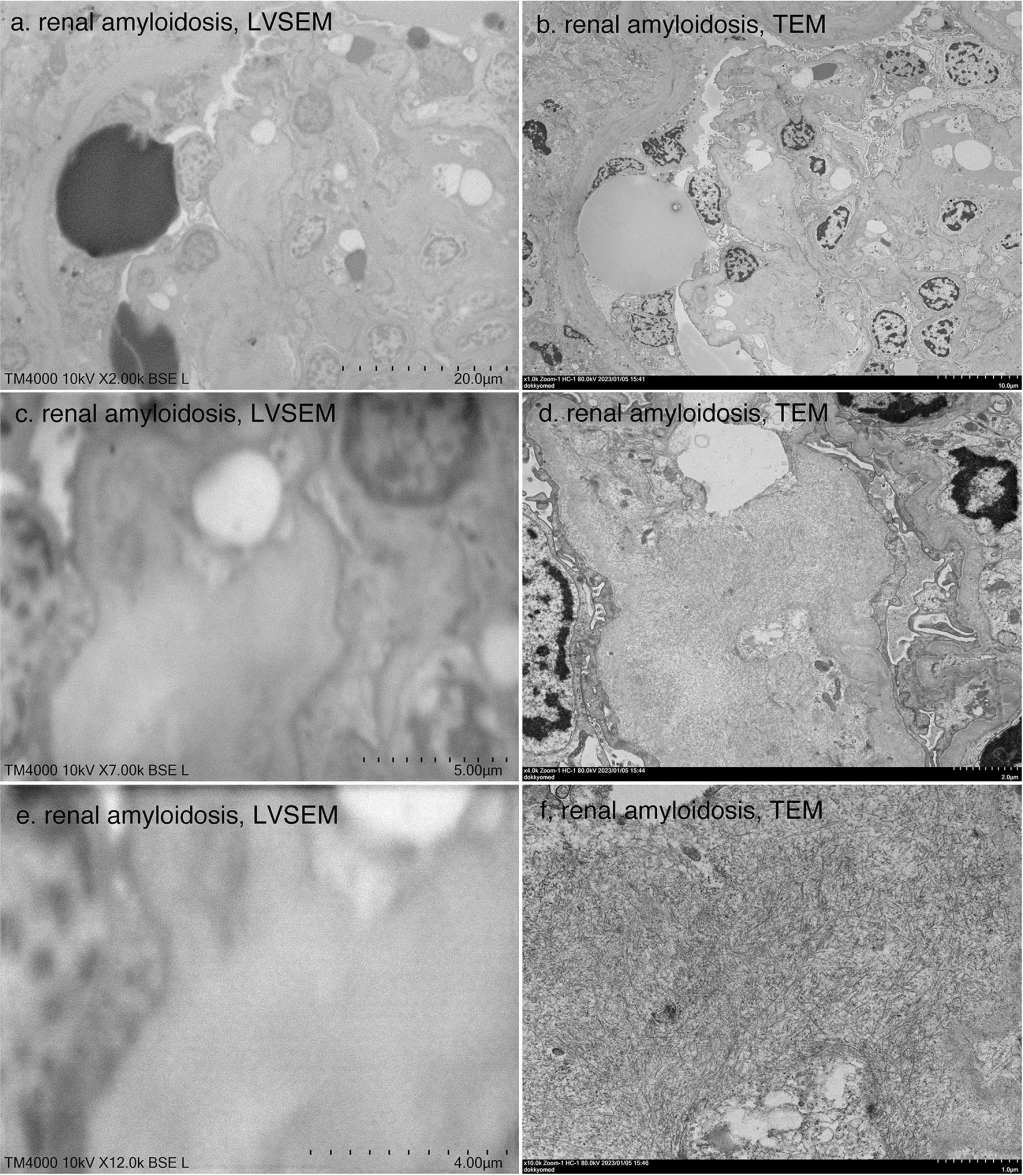
Comparison of LVSEM images of epoxy resin block (**a**, **c**, **e**) and TEM images of ultrathin section (**b**, **d**, **f**) of the same area of renal biopsy samples of renal amyloidosis

In Fabry disease, globotriaosylceramide Gb3-accumulated podocytes can be easily observed at low magnification by LVSEM (Fig. 7a, c) and TEM (Fig. 7b, d). LVSEM was able to observe the entire sample without grids at low magnification, making ceramide identification easier than TEM. In TEM at 40,000 × magnification, a regular zebra pattern was detected in Gb3, confirming zebra bodies (Fig. 7h). However, this fine regular pattern was not detected by LVSEN (Fig. 7g). Renal amyloidosis was also observed by LVSEM as deposits of amorphous low-electron-dense deposits in mesangial regions (Fig. 8a). The identification of the 10 nm amyloid fibrils seen by TEM (Fig. 8b, d, f) was difficult with LVSEM at magnifications between 7000 × and 12,000 × (Fig. 8c, e).

## Discussion

In this study, we first applied LVSEM to directly observe the epoxy resin block to obtain better images of renal biopsy samples. We believe this method will expand the use of LVSEM in clinical laboratories where TEM is not available and aid in the diagnosis of renal biopsies.

### Advantages of LVSEM observation using epoxy resin blocks compared to PAM-stained paraffin sections

LVSEM for use on renal biopsy samples was first introduced by observing PAM-stained paraffin sections as an alternative to TEM [1]. However, cells and organelles were denatured during the processes of dehydration, embedding, and staining with PAM in paraffin sections, resulting in poor preservation of fine structures [1–4]. In contrast, the glutaraldehyde- and osmium-fixed and epoxy resin-embedded specimens for TEM show a clear membrane structure with well-preserved organelles, such as nuclear membranes, mitochondria, and lysosomes. Osmium fixation eliminates the need for PAM staining and electronic stains, such as platinum blue [1] or Ponceau-S solution [6], required for paraffin sections. Recently, LVSEM using an ultravariable pressure detector (UVD) and placing an ultrathin section on a special holder for scanning transmission electron microscopy (STEM) images (UVD-STEM holder LVSEM) has been reported [7]. As the direct observation of epoxy resin blocks by LVSEM in the present study does not require ultrathin sections, it is more convenient than UVD-STEM holder LVSEM.

### Advantages and disadvantages of direct observation of epoxy resin blocks by LVSEM

The method of direct observation of epoxy resin blocks by LVSEM presented in this study is simpler than the observation of ultrathin sections by TEM. It takes several minutes for the glomeruli to appear on the surface of the block cut with a glass knife or diamond knife, but can be seen with a stereomicroscope. Thus, it does not require any technical skill for ultrathin sectioning or mounting on grids. Moreover, electrostaining with uranium acetate and lead citrate is not required, avoiding environmental issues with these chemicals. LVSEM also provides a wide field of view of the entire block, eliminating the problem of visual obstruction by the TEM grids.

Direct observation of epoxy resin block surface is similar to focused ion beam system-scanning electron microscopy (FIB-SEM) and serial block-face scanning electron microscopy (SBF-SEM). However, LVSEM does not require serial cutting of slices with the focused ion beam of the FIB-SEM or the in-scope ultramicrotome of the SBF-SEM, thus preserving renal biopsy specimens. Second, LVSEMs are inexpensive to set up, easy to operate, and can be placed on the benchtop of clinical laboratories. Third, FIB-SEM and SBF-SEM require significant running and maintenance costs by specialized operating technicians, whereas LVSEM can be easily operated by clinicians at any time.

However, a disadvantage of LVSEM is that it takes time to scan the confirmation screen and obtain a clear image to find the target area. It also takes a few minutes to scan and take a picture. Additionally, the resolution is poor at 5000 × or higher. There is unclear identification of the 10 nm amyloid fibrils (Fig. 8) or basement membrane lamellation and reticular formation in Alport syndrome (Fig. 5). Accumulation of ceramide in Fabry's disease can be seen, but zebra structures are not seen at high magnification (Fig. 7). However, if higher magnification images are needed, it is always possible to make ultrathin sections from epoxy resin blocks.

### Usefulness and limitations of LVSEM in renal biopsy diagnosis

Identification of EDD in IgA nephropathy, membranous nephropathy, lupus nephritis, and membranoproliferative glomerulonephritis is fully diagnostic at magnifications up to 5000 × by direct epoxy resin block observation by LVSEM (Figs. 2, 3, 4). The image resolution is superior to LVSEM images of PAM-stained glass slide sections [1, 2] and comparable to the UVD-STEM holder LVSEM method [7]. Foot process changes in the MCNS and basement membrane thickening in the DMN were well diagnosed with direct observation of epoxy resin blocks by LVSEM (Fig. 6). These changes in podocytes of MCNS and podocyte detachment in FSGS were also observed by LVSEM with Ponceau-S-stained PAM sections [8]. We previously reported podocyte albumin transport using immunogold particle labeling of albumin by high-resolution SEM with backscatter electron imaging [9], but LVSEM has resolution limitations in identifying nanogold particles. Gold (III) chloride solution enhances diaminobenzidine staining of plasmalemmal vesicle-associated protein-1 (PV-1) immunohistochemistry in paraffin-embedded sections. LVSEN with this staining showed that PV-1 is overexpressed in glomerular endothelial cells causing oxidative stress accumulation in podocytes in glomerulonephritis with monoclonal IgG deposits [10].

Okada et al. [4] demonstrated the three-dimensional basket-weave appearance of the GBM in Alport syndrome as well as thinning of the GBM in TBMD by PAM-staining LVSEM. The basket-weave appearance of the GBM seems to be difficult to distinguish from the tangential section of the GBM, which shows a mesh structure similar to the basket-weave appearance [4]. Without the help of TEM images, it may be difficult to diagnose lamellation of the GBM in Alport syndrome samples. It is also possible to detect irregular basement membrane thickness in Alport syndrome and GBM thinning in TBMD with direct epoxy resin block observation with LVSEM, but impossible to detect the lamellation of GBM (Fig. 5). The UVD-STEM holder LVSEM images also did not show the features of Alport syndrome [7]. Neither UVD-STEM holder LVSEM [7] nor epoxy resin block LVSEM could detect amyloid fibrils (Fig. 8); thus, further improvement of the resolution of LVSEM itself is necessary to diagnose renal amyloidosis by LVSEM. In this study, we first demonstrated the diagnostic usefulness of direct epoxy resin observation by LVSEM for Fabry disease. Direct observation of epoxy resin blocks by LVSEM, along with light microscopy and immunofluorescence findings, is useful for clinicopathological diagnosis of renal biopsies, except for some renal diseases that require high resolution and magnifications of 10,000 × or higher. The usefulness of LVSEM in renal biopsy compared to TEM is summarized in Table 1.

**Table 1:** Usefulness of LVSEM in renal biopsy compared to TEM

Renal disease	LVSEM	TEM
IgA nephropathy	◯	◯
Membranous nephropathy	Stage I △	◯
	Stage II–III ◯	◯
Lupus nephritis, MPGN	◯	◯
Alport syndrome	△ (lamellation/reticular formation x)	◯ (> × 10,000)
Thin basement membrane	◯	◯
Minimal change nephrotic syndrome	◯	◯
Focal segmental glomerulosclerosis	◯	◯
Diabetic nephrosclerosis	◯	◯
Fabry disease	◯ (fine zebra pattern x)	◯ (> × 20,000)
Amyloidosis	x	◯ (> × 20,000)

## Conclusion

Direct epoxy resin block observation by the LVSEM method is useful for electron microscopic diagnosis in renal biopsy with the same power as TEM at low magnifications of up to 5000 ×.

## Data Availability

All the photos are seen as original images with scal bars and magnifications. There are no numerical data.
